# Development and evaluation of a predictive model based on multi-frequency magnetic resonance elastography for high-risk esophagogastric varices in patients with cirrhotic portal hypertension

**DOI:** 10.1186/s13244-026-02246-z

**Published:** 2026-03-16

**Authors:** Wen Lv, Lu Yu, Liqin Wang, Shuangyan Tang, Jiaxin Yuan, Zhiqiang Wu, Hongjie Cai, Wenbo Guo, Can-hui Sun

**Affiliations:** 1 Department of Radiology, The First Affiliated Hospital, Sun Yat-sen University, Guangzhou, Guangdong People’s Republic of China; 2 Department of Radiology, Jinan University First Affiliated Hospital, Guangzhou, Guangdong People’s Republic of China; 3 Department of Radiology, The Eighth Affiliated Hospital of Sun Yat-sen University, Shenzhen, People’s Republic of China; 4 Department of Interventional Radiology, The First Affiliated Hospital, Sun Yat-sen University, Guangzhou, Guangdong People’s Republic of China

**Keywords:** Multi-frequency magnetic resonance elastography, Esophagogastric varices, Hepatic stiffness, Splenic stiffness, Predictive model

## Abstract

**Objectives:**

This study aimed to assess the severity of esophagogastric varices (EGV) in patients with cirrhotic portal hypertension by measuring liver and spleen stiffness with multi-frequency magnetic resonance elastography (MRE), and to develop a predictive model for identifying high-risk EGV.

**Materials and methods:**

Fifty-four patients with cirrhosis and portal hypertension were enrolled. Clinical and imaging parameters were analyzed, and Bland–Altman analysis and intraclass correlation coefficient (ICCs) evaluated interobserver agreement. Independent-sample *t*-tests were used to analyze the differences in liver and spleen stiffness between high-risk and low-risk groups. The correlations between variables and the endoscopic EGV severity were analyzed by Spearman’s correlation analysis and univariate logistic analysis. Multivariate logistic regression identified independent predictors, and receiver operating characteristic (ROC) and decision curve analyses assessed model performance and clinical utility. Bootstrap resampling and sensitivity analyses validated model robustness.

**Results:**

Bland–Altman analysis and ICC analysis showed high consistency. There were significant differences in liver and spleen stiffness between low-risk and high-risk patients. Platelet count and plateletcrit were negatively correlated with variceal severity, whereas bilirubin, INR, liver/spleen stiffness, spleen volume, and portal/splenic vein diameters showed positive correlations. Multivariate analysis identified spleen stiffness and portal vein diameter as independent predictors. The predictive model *y* = −17.50 + 2.77× spleen stiffness +0.57× portal vein diameter demonstrated superior predictive performance compared to individual indicators. Bootstrap resampling and sensitivity analyses demonstrated the robustness and stability of the model’s predictive performance.

**Conclusion:**

The model effectively identifies high-risk EGV, highlighting the potential of noninvasive MRE-based assessment to guide early intervention and reduce variceal bleeding risk.

**Critical relevance statement:**

The predictive model based on multi-frequency MRE and MRI parameters (spleen stiffness + portal vein diameter) can effectively screen out high-risk EGV, which is helpful for guiding timely clinical intervention, so as to reduce the risk of EGV bleeding in these patients, and improve their prognoses.

**Key Points:**

- Multifrequency magnetic resonance elastography (MRE) can indicate the stiffness of the liver and spleen.
- The splenic stiffness is a reliable predictor of high-risk EGV.
- The predictive model can effectively screen out high-risk EGV.

**Graphical Abstract:**

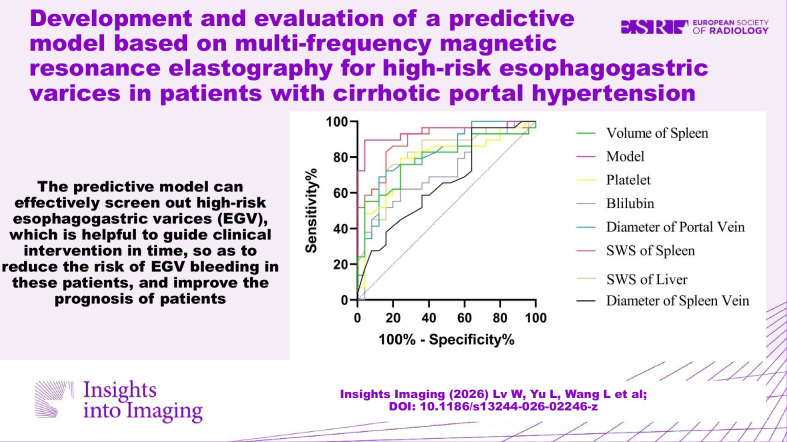

## Introduction

Esophagogastric varices (EGV) are a common complication of decompensated cirrhotic portal hypertension, with an incidence rate as high as 85% in patients with decompensated cirrhosis [1]. Esophagogastric variceal bleeding (EVB) is the main cause of death for patients with liver cirrhosis, with a 1-year incidence of approximately 5–15% and a 6-week mortality rate of 10%–20% once bleeding occurs [2, 3]. Therefore, early identification of high-risk EGV and timely intervention are crucial for improving the prognosis of patients with portal hypertension.

To date, endoscopy is regarded as the “gold standard” for diagnosing the presence of varices and predicting bleeding risk [4]. Baveno VI recommends that all patients undergo an upper gastrointestinal endoscopy upon diagnosis of cirrhosis [5]. Endoscopy, however, is an invasive procedure that can cause discomfort and carries potential risks such as anesthesia-related complications and bleeding, limiting its usage in routine monitoring.

Previous studies have shown that noninvasive methods, including platelet count (PLT), Aspartate Aminotransferase to Platelet Ratio Index (APRI), liver and spleen volume, vascular diameter or volume can be used for diagnosing EGV and predicting high-risk groups [6–10]. However, clinical application of these methods is influenced by insufficient diagnostic accuracy or inconsistent cutoff values. Therefore, identifying other non-invasive indicators to predict high-risk EGV is of great importance.

Magnetic resonance elastography (MRE), known as an “imaging palpation technique”, allows for the quantitative assessment of tissue elasticity or stiffness in living organisms [11–13]. Growing evidence suggests that liver and spleen stiffness measured by MRE are capable of predicting the risk of EGV [14–17], but most of these studies utilized traditional single-frequency MRE [14–16, 18].

The multifrequency MRE technique is an emerging noninvasive imaging modality used to characterize the biomechanical properties of the tissue. Although few head-to-head studies have quantitatively compared multi- and single-frequency MRE in the same population, existing technical evidence indicates that multi-frequency MRE offers potential advantages. It involves multiple frequencies combined with the k-MDEV algorithm, providing enhanced noise robustness and tissue uniformity and more anatomical details (Fig. S1) compared to single-frequency methods [17]. Additionally, four passive drivers enable the vibration waves of multi-frequency MRE to be effectively transmitted to the deep areas of the organs, which is beneficial for patients with splenomegaly, massive ascites or obesity [19]. However, a study has reported that the absolute values of liver and spleen stiffness at different frequencies and their correlations with portal hypertension and high-risk EGV are different [20]. The predictive value of multi-frequency MRE for high-risk EGV is still unclear. At present, there are relatively few relevant studies exploring the potential of multifrequency MRE in the diagnosis of EGV.

Therefore, this study aims to assess the severity of EGV in patients with cirrhotic portal hypertension by measuring liver and spleen stiffness with multi-frequency MRE, and to preliminarily develop a predictive model for identifying high-risk EGV based on multi-frequency MRE and other clinical and imaging information.

## Materials and methods

### Participants

**Fig. 1:**
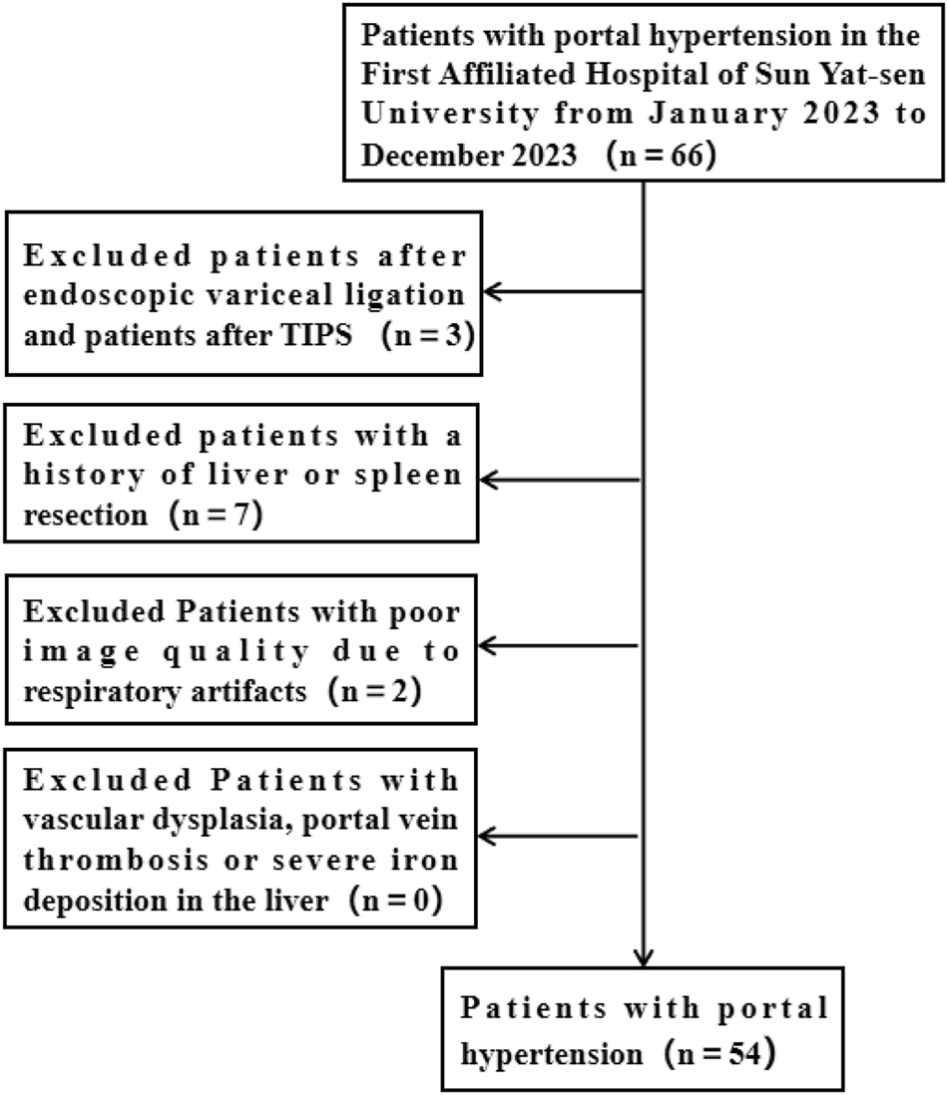
Inclusion and exclusion process of volunteers

The Ethics Committee of the First Affiliated Hospital of Sun Yat-sen University approved this prospective study (approval No. 2023-590). From January to December 2023, 66 patients were screened according to the following inclusion criteria: (1) Aged ≥ 18 years; (2) Patients with hepatitis-related cirrhosis and portal hypertension confirmed by the clinical, radiologic, and laboratory findings based on the Chinese Society of Hepatology (2019) [21]. Detailed criteria were provided in the supplementary materials. Exclusion criteria were: (1) Poor imaging quality due to respiratory motion artifacts (*n* = 2); (2) Prior biopsy, chemotherapy, or other treatments before MRE (*n* = 10); (3) Incomplete clinical information and MRE data (*n* = 0); (4) Vascular abnormalities, portal vein thrombosis or severe iron deposition in the liver (*n* = 0). Finally, 54 patients were enrolled (Fig. 1). Written informed consent was obtained from all participants prior to their enrollment.

### MR techniques

#### MR sequences

All MR examinations were conducted with a 3.0-Tesla imaging system (MAGNETOM Prisma; Siemens Healthcare) equipped with 18-channel phased-array surface coils. To avoid the confounding effects of overestimated stiffness values, all volunteers were required to fast for ≥ 6 h before the examination [16]. Detailed MRI and multi-frequency MRE acquisition protocols are provided in the Supplementary Methods. Table 1 lists the MRE sequence parameters. All the sequences, except elastrography, were designed to screen for liver and spleen disease.

**Table 1:** Sequence parameters of the liver and spleen magnetic resonance imaging protocol

Sequence	Contmre-Tra	VIBE-Q-Dixon	T2-Haste-Cor	T2-Trigger-Tra
Repetition time/echo time (ms)	1780/42	9/1.05, 2.46, 3.69, 4.92, 6.15, 7.38	1000/70	2000/77
Flip angle (°)	90	4	160	93
Section thickness (mm)	4	2	5	5
Field of view (mm)	420	426	400	380
Acquisition time (s)	198	20	31	255
Acquisition matrix	130 × 168	111 × 160	168 × 256	288 × 384
Acceleration factor	2	4	3	3
Receiving bandwidth (Hz/Px)	2480	1080	1221	766

*cor* coronal, *tra* transverse, *VIBE* volume-interpolated breath-hold

#### Imaging process and liver and spleen stiffness and spleen volume measurements

We processed the multi-frequency wave field data using the pipeline at https://bioqic-apps.com. Full field-of-view maps of shear wave speed (SWS; *c*, m/s) were generated using a multi-frequency wave number-based processing algorithm (k-MDEV) and a Laplacian operators-based processing method (MDEV) [17]. SWS was known as a surrogate marker of stiffness, reflecting the magnitude of the complex shear modulus [22].

**Fig. 2:**
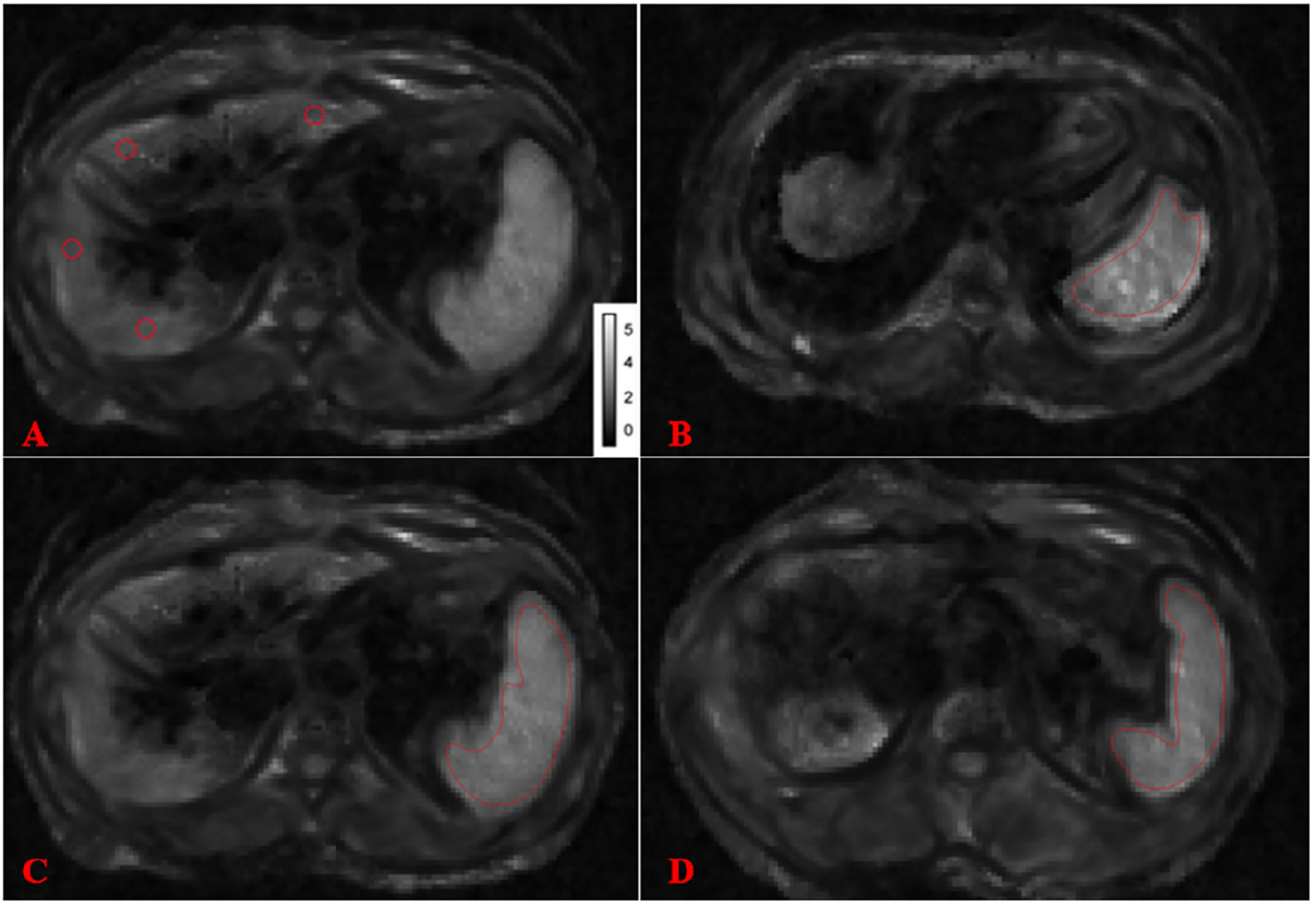
Examples of the regions of interest (ROIs) used to measure liver and spleen stiffness. ROI used to measure liver stiffness (**A**) and spleen stiffness in the upper (**B**), middle (**C**), and lower (**D**) slices. MRE was performed using four compressed air-driven pressure pads (0.6 bar amplitude) with motion encoding in three directions and 2D inversion. Wave fields were sampled at four mechanical frequencies (30, 40, 50, and 60 Hz); Motion-encoding gradient (MEG) frequency = 69.44 Hz, amplitude = 45 mT/m

Two radiologists with 10 years (reader 1) and 2 years (reader 2) of experience with hepatic and splenic MRE independently analyzed the images using ImageJ software (version 1.51), blinded to the patients’ information. Manually drawn regions of interest (ROIs) (about 1.0 cm in diameter) were positioned in the most homogenous part of each segment of the liver parenchyma (portal vein-level slice), and then perform the same operation on the upper and lower slices (Fig. 2) [23]. For the spleens, ROIs were placed at the upper, middle and lower slices, including an entire cross-sectional image of the spleen parenchyma (Fig. 2). Large vessels and calcification were avoided. SWS values were averaged to obtain the mean hepatic or splenic SWS.

For spleen volume, reader 2 imported 5-mm images into the Volume workstation and manually delineated splenic margins layer by layer, avoiding the splenic hilum. The volume was automatically calculated by the workstation.

### Clinical and other imaging data Collection

**Fig. 3:**
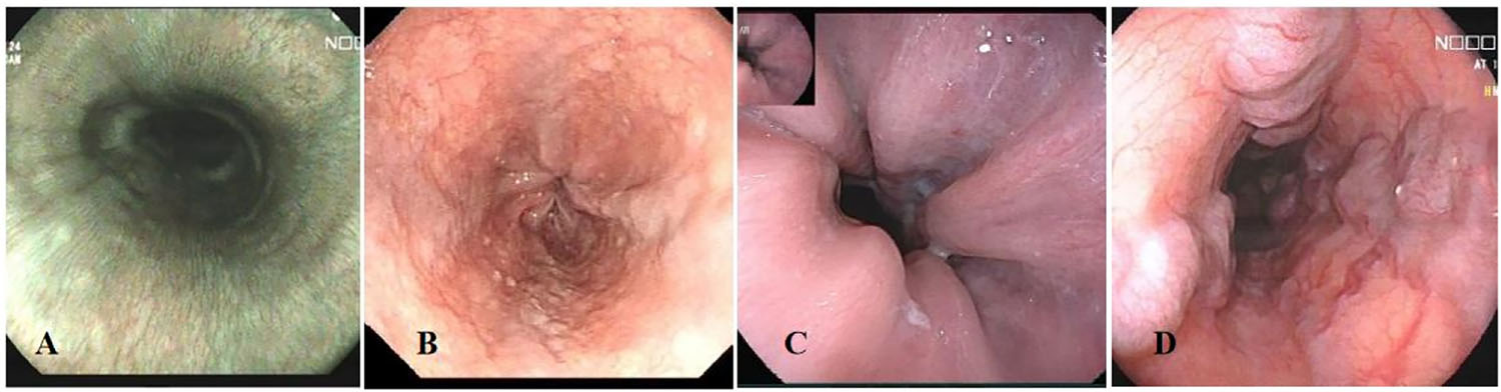
Endoscopic grading of esophagogastric varices. **A** G0: without esophagogastric varices; **B** G1: mild esophagogastric varices; **C** G2: moderate esophagogastric varices; **D** G3: Severe esophagogastric varices. Images were obtained during routine upper endoscopy in patients with hepatitis-related cirrhosis and portal hypertension

Upper gastrointestinal endoscopy was performed by 2 gastroenterologists with 10 or more years of endoscopic experience. EGVs were classified as follows (Fig. 3): (1) G0: Without EGVs; (2) G1: EGVs are straight or mildly tortuous; (3) G2: EGVs are straight or mildly tortuous with the red sign, or serpentine varices with dilation and tortuosity without the red sign; (4) G3: EGVs run obliquely and are tortuous, with a tumorlike appearance [15]. Patients were divided into two groups—a low-risk group with no or small varices (G0 or 1) and a high-risk group with large varices (G2 or 3)—on the basis of the probability of developing EVB [15]. In cases of disagreement, the final grade was determined by consensus after joint review and discussion to ensure accuracy and consistency.

The following clinical parameters and serum markers tests were retrieved from the electronic medical record by a clinician who was blind to imaging and endoscopy information: age, sex, platelet count (PLT), platelet packed cell volume (PCT), total bilirubin, serum albumin, prothrombin time (PT), international normalized ratio (INR), serum aspartate aminotransferase (AST), serum alanine aminotransferase (ALT) and the amount of ascites. Liver function was assessed for each patient according to the Child-Pugh score.

Portal and splenic vein diameters were measured on axial contrast-enhanced images using a standardized protocol (Supplementary Material).

### Statistical analyses

Continuous variables are expressed as means ± standard deviation. Categorical variables are expressed as proportions or percentages. Interobserver agreements were assessed using Bland–Altman analysis and intraclass correlation coefficients (ICCs). Independent-sample *t*-tests were used to analyze the differences in liver and spleen stiffness between high-risk and low-risk groups. The correlations between each factor and the severity of EGV under endoscopy were analyzed by Spearman’s correlation analysis and univariate logistic analysis, and then ROC curves were drawn. A binary logistic regression analysis was performed to develop a predictive model, and ROC curves were also generated. Delong’s test was used to compare the predictive performance of the model with that of individual factors and a guidance-based model. Decision curve analysis was used to evaluate clinical efficacy. To assess the internal robustness of the predictive model and mitigate potential overfitting, internal validation was performed using bootstrap resampling. And sensitivity analysis was performed by excluding extreme outliers of spleen stiffness and portal vein diameter using the interquartile range (IQR) method. The statistical analyses were performed using MedCalc software (MedCalc Software, Ltd, version 20.218). A two-sided *p* < 0.05 indicated a statistically significant difference.

## Results

### The characteristics of patients

**Fig. 4:**
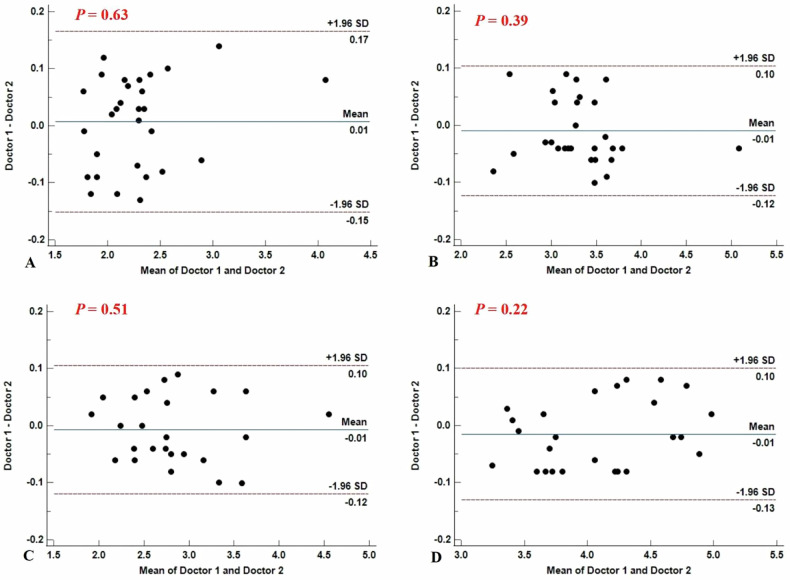
Bland–Altman analyses of interobserver agreement. **A** Bland–Altman analysis for liver stiffness measurement in the low-risk group; **B** Bland–Altman analysis for spleen stiffness measurement in the low-risk group; **C** Bland–Altman analysis for liver stiffness measurement in the high-risk group; **D** Bland–Altman analysis for spleen stiffness measurement in the high-risk group

A total of 54 patients with cirrhosis and portal hypertension were included in this study (46 males, 8 females, average age: 54.52 ± 9.65 years, patients with Child-Pugh grade A: 48; patients with Child-Pugh grade B: 4; patients with Child-Pugh grade C: 2; patients with grade G0: 21; patients with grade G1: 8; patients with grade G2: 12; patients with grade G3: 13). Based on the risk of EVB, patients with grade G0 and G1 were included in the low-risk group, and patients with grade G2 and G3 were included in the high-risk group [24]. In the low-risk group, liver SWS measured by doctors 1 and 2 was 2.28 ± 0.47 and 2.27 ± 0.45 m/s, respectively, and spleen SWS was 3.30 ± 0.48 and 3.31 ± 0.49 m/s. In the high-risk group, liver SWS was 2.83 ± 0.58 and 2.83 ± 0.59 m/s, and spleen SWS was 4.07 ± 0.52 and 4.09 ± 0.51 m/s. Interobserver agreement for liver and spleen shear wave stiffness measurements was excellent, with ICCs of 0.99 for both organs in the low- and high-risk groups (95% CIs shown in Table 2; all *p* < 0.001), consistent with the Bland–Altman analysis (Fig. 4). Finally, the average value of the two measurements was taken and recorded. The mean values of SWS of liver and spleen in the low-risk group were 2.28 ± 0.46 m/s and 3.31 ± 0.48 m/s, respectively. In the high-risk group, the mean liver stiffness was 2.83 ± 0.59 m/s, and the mean spleen stiffness was 4.08 ± 0.52 m/s. There were significant differences in liver and spleen stiffness between low-risk patients and high-risk patients (*p* < 0.05).

**Table 2:** Interobserver agreement for Liver and spleen measurement

	SWS of the liver in the low-risk group	SWS of the liver in the high-risk group	SWS of the spleen in the low-risk group	SWS of the spleen in the risk group
ICC	0.99	0.99	0.99	0.99
95% CI	0.984–0.996	0.995–0.999	0.993–0.998	0.993–0.999
*p*-value	< 0.001	< 0.001	< 0.001	< 0.001

*CI* confidence interval, ≤ 0.20, poor agreement; 0.21–0.40, fair agreement; 0.41–0.60, moderate agreement; 0.61–0.80, substantial agreement; and > 0.80, near perfect agreement

### Predictors of high-risk EGV

**Fig. 5:**
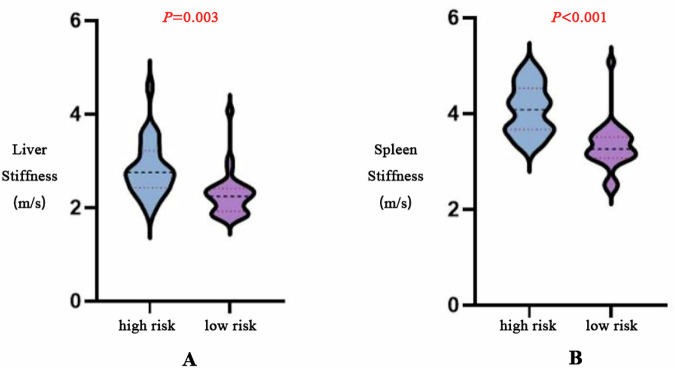
Violin diagram of liver (**A**) (2.83 ± 0.58 m/s vs. 2.28 ± 0.47 m/s) and spleen (**B**) (4.07 ± 0.52 m/s vs. 3.30 ± 0.48 m/s) stiffness distribution in high and low risk patients

The average level of AST in the low-risk group was 43.86 ± 21.93 U/L, and the average level of AST in the high-risk group was 62.72 ± 86.09 U/L. The average ALT level of the low-risk group was 35.62 ± 22.02 U/L, and that of the high-risk group was 49.28 ± 74.67 U/L. The average albumin level of the low-risk group was 40.94 ± 7.63 g/L, and that of the high-risk group was 38.00 ± 9.42 g/L; The average bilirubin level of the low-risk group was 20.71 ± 8.66 µmol/L, and that of the high-risk group was 36.96 ± 50.18 µmol/L; The average PLT level of the low-risk group was 116.07 ± 49.88 × 10^9^/L, and that of the high-risk group was 69.24 ± 35.35 × 10^9^/L. The average PCT level of the low-risk group was 0.11 ± 0.05, and the average PCT level of the high-risk group was 0.07 ± 0.04. The average INR value of the low-risk group was 1.07 ± 0.14. The average INR value of the high-risk group was 1.20 ± 0.16. The mean spleen volume was 456.63 ± 301.45 mL in the low-risk group and 752.09 ± 240.46 mL in the high-risk group. The mean portal vein diameter was 11.61 ± 1.59 mm in the low-risk group and 14.38 ± 2.52 mm in the high-risk group. The mean splenic vein diameter was 8.33 ± 1.94 mm in the low-risk group and 9.82 ± 2.63 mm in the high-risk group. Figure 5 shows the violin diagram of the distribution of liver and spleen stiffness in high and low-risk patients.

### Prediction performance of clinical indicators, imaging indicators and the combined model

Spearman’s correlation analysis and univariate logistic analysis showed that age, sex, serum AST level, serum ALT level, serum albumin level, and Child-Pugh grade were not associated with the degree of EGV under endoscopy (*p* > 0.05). PLT (*r* = −0.49, 95% confidence interval (CI): −0.67 to 0.26, cut-off value: 78.00 × 109/L, AUC: 0.78, sensitivity (Se): 0.76, specificity (Sp): 0.79), PCT (*r* = −0.40, 95%CI: −0.61 to 0.15, cut-off value: 0.09, AUC: 0.73, Se: 0.84, Sp: 0.62) were negatively correlated with the degree of EGV under endoscopy (*p* < 0.05). Bilirubin (*r* = 0.36, 95%CI: 0.11–0.57, cut-off value: 17.70 µmol/L, AUC: 0.71, Se: 0.88, Sp: 0.52), INR (*r* = 0.46, 95%CI: 0.21–0.65, cut-off value: 1.03, AUC: 0.76, Se: 0.92, Sp: 0.55), liver stiffness (*r* = 0.54, 95%CI: 0.32–0.71, cut-off value: 2.38 m/s, AUC: 0.81, Se: 0.84, Sp: 0.72), spleen stiffness (*r* = 0.67, 95%CI: 0.49–0.80, cut-off value: 3.38 m/s, AUC: 0.89, Se: 0.84, Sp: 0.83), spleen volume (*r* = 0.50, 95%CI: 0.26–0.68, cut-off value: 568.18 mL, AUC: 0.79, Se: 0.78, Sp: 0.75), portal vein diameter (*r* = 0.55, 95% CI: 0.33-0.71, cut-off value: 11.8 mm, AUC: 0.82, Se: 0.88, Sp: 0.68), splenic vein diameter (*r* = 0.28, 95% CI: 0.01–0.51, cut-off value: 10.7 mm, AUC: 0.66, Se: 0.36, Sp: 0.97) were positively correlated with the degree of EGV under endoscopy (*p* < 0.05). The relevant data are shown in Table 3.

**Table 3:** Spearman’s correlation coefficient, univariate logistic regression and ROC curve results of relevant clinical and imaging indicators

	*p*-value	*r*	95% CI	Cut off value	AUC	Sensitivity	Specificity
Age	> 0.05	-	-	-	-	-	-
Sex	> 0.05	-	-	-	-	-	-
AST	> 0.05	-	-	-	-	-	-
ALT	> 0.05	-	-	-	-	-	-
Albumin	> 0.05	-	-	-	-	-	-
Child-Pough classification	> 0.05	-	-	-	-	-	-
PLT	**< 0.05**	−0.49	−0.67 to −0.26	78.00 × 10^9^/L	0.78	0.76	0.79
PCT	**< 0.05**	−0.40	−0.61 to −0.15	0.09	0.73	0.84	0.62
Bilirubin	**< 0.05**	0.36	0.11–0.57	17.70 µmol/L	0.71	0.88	0.52
INR	**< 0.05**	0.46	0.21–0.65	1.03	0.76	0.92	0.55
SWS of liver	**< 0.05**	0.54	0.32–0.71	2.38 m/s	0.81	0.84	0.72
SWS of Spleen	**< 0.05**	0.67	0.49–0.80	3.38 m/s	0.89	0.84	0.83
Volume of Spleen	**< 0.05**	0.50	0.26–0.68	568.18 mL	0.79	0.78	0.75
Diameter of Portal Vein	**< 0.05**	0.55	0.33–0.71	11.8 mm	0.82	0.88	0.69
Diameter of Spleen Vein	**< 0.05**	0.28	0.01–0.51	10.7 mm	0.66	0.36	0.97

*ALT* glutamic pyruvic transaminase, *AST* glutamic oxaloacetic transaminase, *INR* International normalized ratio, *AUC* area under the curve, 95% CI 95% confidence interval

*p* < 0.05 was considered statistically significant

Bold values indicate statistical significant results

Indicators that were significant in univariate analyses were incorporated into the multivariate logistic regression model. Multivariate logistic regression analysis showed that spleen stiffness and portal vein diameter were independent predictors of the degree of EGV under endoscopy. The predictive model was *y* = −17.50 + 2.77× spleen stiffness (m/s) +0.57× portal vein diameter (AUC = 0.94, 95%CI: 0.83–0.98, boundary value: −0.93, Se: 0.80, Sp: 0.97).

**Fig. 6:**
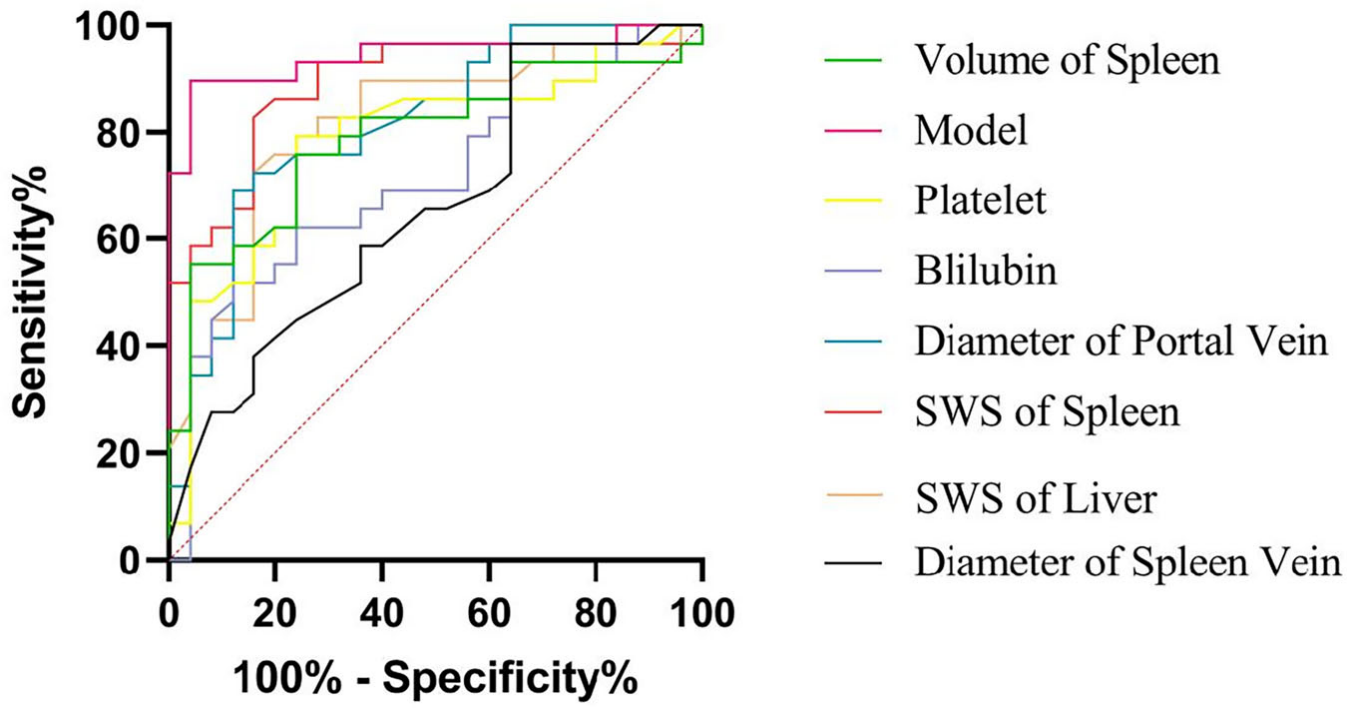
Receiver operating characteristic (ROC) curve analysis of clinical indicators, imaging indicators, and the combined predictive model for identifying high-risk esophagogastric varices. In this single-center cohort, the combined model demonstrates the highest discriminative performance (AUC = 0.94) compared with individual clinical and imaging indicators (all *p* < 0.05). The predictive model should be interpreted as exploratory and requires independent external validation before clinical application

Delong’s test showed that the prediction efficiency of the predictive model was higher than any of the clinical and imaging indicators, and the difference was statistically significant (*p* < 0.05). The ROC curves are shown in Fig. 6. The decision curve is shown in Fig. S2. Bootstrap internal validation demonstrated a mean AUC of 0.92 (95% CI 0.765–1) (Fig. S4). Sensitivity analysis was performed by excluding extreme outliers of spleen stiffness (m/s) and portal vein diameter using the interquartile range (IQR) method. After refitting the model, the regression coefficients and AUC values remained similar to those of the original model (AUC before vs. after exclusion: 0.94 vs. 0.93) (Fig. S5).

### Comparison with the Baveno VII–based model

To facilitate direct comparison, we constructed a Baveno VII-based model using liver stiffness and PLT within our cohort [25]. This model achieved an AUC of 0.90 (95% CI: 0.83–0.96), Se = 0.84, and Sp = 0.83, with a bootstrap-corrected AUC of 0.88 (95% CI: 0.74–0.99) (Fig. S6). The Delong’s test showed no statistically significant difference between the two models (*p* = 0.30) (Fig. S7).

## Discussion

Non-invasive prediction of high-risk EGV is important to screen patients who need gastroscopy, consistent with contemporary precision medicine, and potentially enhancing patient adherence.

In this study, age, sex, serum AST level, serum ALT level, serum albumin level, and Child-Pugh grade were not associated with EGV severity. PLT and PCT were negatively correlated, while bilirubin, INR, liver stiffness, spleen stiffness, spleen volume, portal vein diameter and splenic vein diameter were positively correlated. Spleen stiffness and portal vein diameter were independent predictors, and the model combining both outperformed single factors in predictive efficacy and specificity.

Decreased PLT, increased albumin and prolonged PT were common in liver cirrhosis, with about 84% of patients showing thrombocytopenia [26]. Several studies reported that PLT, albumin, and INR could predict high-risk EGV: more severe EGV was associated with lower PLT and PCT, higher albumin, and longer PT [26–30]. Baveno VI suggested that patients with liver stiffness < 20 kPa and PLT > 150 × 10^9^/L may not require gastroscopy [5]. However, cut-off values of PLT, albumin and INR varied across studies, with limited diagnostic efficacy and specificity, likely because these markers mainly reflect liver function [29]. And there was still no sufficient evidence that the level of liver function was related to portal vein pressure [18]. Thus, some patients may be missed using these factors, and their predictive value may differ in portal hypertension with or without cirrhosis, warranting further large-scale studies.

The results of our study indicated that spleen stiffness was an independent predictor of the degree of EGV and was positively correlated. This may be due to the consequent changes in the hemodynamics and structure of the spleen in patients with portal hypertension, including increased splenic blood flow, hyperplasia of red pulp, and fibrosis, all of which lead to increased spleen stiffness [31, 32]. Therefore, it was feasible to predict high-risk EGV by reflecting the level of portal pressure by spleen stiffness, which was also consistent with the results of several previous studies [15, 18, 20]. In this study, portal vein diameter was also an independent predictor of the degree of EGV, with a positive correlation. In addition, splenic vein diameter was positively correlated with the extent of the esophagogastric fundal vein. This may be due to the increase in portal vein pressure in patients with portal hypertension, and the increase of portal vein and splenic vein blood flow, resulting in the increase of portal vein and splenic vein diameter [33].

The liver stiffness was positively correlated with the degree of EGV under endoscopy. The higher the stiffness of the liver, the more severe the degree of EGV under endoscopy, which is consistent with the results of previous studies [14–16]. However, the predictive efficacy of this indicator is lower than that of spleen stiffness. Romot M et al showed that spleen stiffness had a higher predictive value for high-risk EGV than liver stiffness, which was consistent with the results of this study [20]. This may be because liver stiffness mainly reflected the level of portal vein pressure through the level of liver fibrosis [16]. Some studies have shown that portal hypertension developed before or after liver fibrosis, which may mean that the level of portal vein pressure did not exactly correspond to the severity of liver fibrosis, and the spleen stiffness may more directly reflect the level of portal vein pressure [16, 33, 34]. Some other studies believe that this is because liver stiffness will stop changing when the portal vein pressure increases to a certain extent, while the spleen stiffness has been continuously changing after the change of PV [35–37]. Therefore, compared with the Baveno VII criterion that recommends liver stiffness less than 20 kPa and PLT more than 150 × 10^9^/L for distinguishing high-risk EGV, we believe that the model based on spleen stiffness is more reliable.

The volume of spleen was positively correlated with the degree of EGV under endoscopy, which was consistent with the findings of Morisaka H et al [16]. This may be due to the increased splenic blood flow in patients with portal hypertension, which leads to red pulp hyperplasia, thus increasing spleen volume [38]. However, some studies have shown that spleen volume/length diameter is not related to the degree of gastric varicose veins [14, 15, 18]. It may be due to the large difference in spleen volume between different individuals. For example, some patients originally had a small spleen volume, but in portal hypertension, the spleen volume increased, still within the normal range of spleen volume. In the case of a small experimental sample size, this led to differences in experimental results. The changes in spleen volume before and after the lesion may be more significant, but further studies are needed to confirm this.

In our study, a predictive model was constructed based on the portal vein diameter and spleen stiffness. The results demonstrated that the prediction efficiency of this model was higher than any of the above clinical and imaging indicators, and the difference was statistically significant. Compared with the model based on single-frequency MRE of previous research and criteria of Baveno VII, our model has higher specificity (97%) while ensuring sensitivity (80%) [5, 37]. The high specificity suggests that a substantial proportion of patients could safely avoid unnecessary endoscopic examinations, thereby increasing clinicians’ confidence in adopting a non-invasive management strategy. In direct comparison with the guidance-based model, our model also tended to achieve higher specificity and AUC, although the difference did not reach statistical significance. These findings suggest a potential advantage of our approach, which warrants further validation in larger, multicenter cohorts.

And the decision curve analysis demonstrated that the predictive model provided a greater standardized net benefit than both the “treat-all” and “treat-none” strategies across a threshold probability range of approximately 0.05–0.35. This indicates that applying the model in clinical practice could improve decision-making by identifying high-risk patients who may benefit from prophylactic endoscopic therapy or closer monitoring, while reducing unnecessary procedures in low-risk individuals.

Moreover, internal bootstrap validation demonstrated consistent discriminative performance, supporting the reliability of the model in this cohort. Sensitivity analysis excluding extreme outliers of spleen stiffness and portal vein diameter yielded nearly identical regression coefficients and AUC values, indicating that the model’s predictive ability was not driven by a small number of extreme data points. Collectively, these findings suggest that the model exhibits good internal robustness and stability. Despite the favorable predictive performance, the relatively small sample size of this single-center cohort inevitably increases the risk of model overfitting. Therefore, the proposed model should be considered preliminary and exploratory, and the high AUC may partially reflect optimistic performance estimation. Independent external validation in larger cohorts is mandatory before clinical application.

On the other hand, the measurement of spleen stiffness and portal vein diameter was convenient, and it was feasible to popularize in the clinic. Therefore, the predictive model based on the spleen stiffness measured by multi-frequency MRE combined with the inner diameter of the portal vein could screen out high-risk EGV, thus suggesting timely clinical intervention, reducing the risk of EVB, and helping to improve the prognosis of patients.

Compared to gastroscopy and HVPG, elastography adds only one MRI sequence and can be performed during routine scans, which improves patient compliance. Moreover, MRE poses lower risks, benefiting patients contraindicated for gastroscopy and HVPG. Compared to ultrasound elastography, studies demonstrate that MRE exhibits higher accuracy than ultrasound elastography in predicting high-risk EGV [37, 39, 40]. Additionally, MRE is less affected by ascites and abdominal fat, shows lower operator dependency, and allows for a more comprehensive abdominal assessment [15].

In patients with cirrhotic portal hypertension, accurate assessment of spleen stiffness is technically challenging because of respiratory motion, ascites, obesity, and marked splenomegaly, all of which may compromise wave propagation and measurement stability in conventional single-frequency MRE. Compared to single-frequency MRE, multi-frequency MRE improves noise robustness, tissue uniformity, and detail resolution, enhancing differentiation of parenchymal vs. non-parenchymal liver tissues. Its four passive drivers effectively transmit vibrations deep into organs, benefiting patients with splenomegaly, massive ascites, or obesity. Therefore, the application of multi-frequency MRE in predicting EGV is accurate, stable and reliable, and has the potential for further clinical application.

However, direct head-to-head studies are still needed to confirm these advantages in clinical populations. Multi-frequency MRE also has its limitations. It may lead to underestimation in patients with severe iron deposition [15], indicating the need for further research on such patients.

The study still has the following limitations: (1) Due to the limitations of clinical data, the study failed to explore the continuous changes of spleen volume and liver and spleen stiffness during the progression of EGV, and further research is needed; (2) We did not further distinguish the types of EGV for stratified analysis; (3) The sample size is relatively small to conduce subgroup analysis, and the predictive model needs to be further tested by an external validation set; (4) The ROC-derived cut-off values for spleen stiffness, portal vein diameter, and other indicators were established based on a single-center cohort. These cut-offs may not be directly generalizable to other populations, and external validation in independent cohorts is needed before clinical implementation; (5) Exclusion of patients with prior EVL/TIPS and poor image quality may have introduced spectrum bias, leading to a cohort with relatively better imaging quality and more stable hemodynamics, and potentially a slight overestimation of model performance. To minimize this effect, uniform imaging and measurement protocols were applied, and consecutive eligible patients were enrolled. Future multicenter studies including technically challenging cases are warranted to further verify the model’s robustness and generalizability.

## Conclusion

The predictive model based on multi-frequency MRE and MRI parameters (spleen stiffness + portal vein diameter) demonstrated good performance in identifying patients at high risk of EGV. These preliminary findings suggest a potential role for this noninvasive approach in guiding early intervention to reduce the risk of variceal bleeding. Further validation in larger, multicenter cohorts is needed to confirm its clinical applicability.

## Supplementary information

ELECTRONIC SUPPLEMENTARY MATERIAL

## Data Availability

The original data generated in this study can be requested from the corresponding author. For any researchers interested in obtaining these data, we encourage you to contact the corresponding author via email at sunch@mail.sysu.edu.cn to discuss the specific conditions for data access and possible measures to protect privacy.

## Abbreviations

ALT: Serum alanine aminotransferase
AST: Serum aspartate aminotransferase
AUC: Area under the curve
CI: Confidence interval
EGV: Esophagogastric varices
EVB: Esophagogastric variceal bleeding
ICCs: Intraclass correlation coefficients
INR: International normalized ratio
MRE: Magnetic resonance elastography
PCT: Platelet packed cell volume
PLT: Platelet count
PT: Prothrombin time
ROI: Regions of interest
Se: Sensitivity
Sp: Specificity
SWS: Shear wave speed
