# Supplementary material for: Development and evaluation of a predictive model based on multi-frequency magnetic resonance elastography for high-risk esophagogastric varices in patients with cirrhotic portal hypertension
Source: Insights Imaging. 2026 Mar 16;17:69. doi: 10.1186/s13244-026-02246-z (PMC12992754; doi:10.1186/s13244-026-02246-z)
Supplement: Supplementary file 1 — ELECTRONIC SUPPLEMENTARY MATERIAL [file 13244_2026_2246_MOESM1_ESM.pdf]

Development and Evaluation of a Predictive Model based on Multi-frequency Magnetic Resonance Elastography for High-Risk Esophagogastric Varices in Patients with Cirrhotic Portal Hypertension

ELECTRONIC SUPPLEMENTARY MATERIAL

Insights Imaging (2026) Lv W, Yu L, Wang L, et al.

A: Supplementary Text

Material and Methods

Participants

Detail diagnostic criterias

① Patients who have underwent HVPG measurement, and had HVPG≥10 mmHg, with non-cirrhotic portal hypertension excluded through subsequent evaluations;

②Patients with visible esophagogastric varices confirmed by endoscopy, and non-cirrhotic causes excluded；

③ For patients without HVPG or esophagogastric varices detected by endoscopy in our study, experienced hepatologists (≥10 years of clinical practice) diagnosed portal hypertension based on a combination of the following: a Clinical signs indicative of portal hypertension (e.g., portal hypertensive gastropathy or enteropathy); b. Radiological features on CT or MRI, including splenomegaly, ascites (excluding other causes), or varices in the gastrointestinal tract; c. Platelet count < 110×10^9/L. Patients diagnosed with portal hypertension after comprehensive consideration by clinicians were initially included in this study. In the subsequent MRE examination, the liver stiffness of such patients needed to be greater than 15kPa before they would be finally included in the study.

MR techniques.

MR sequences

The volunteers were positioned in a supine position within the imaging system, with four small plastic driver pads placed on the anterior (two pads) and posterior (two pads) body wall areas over the liver and spleen. The standard baseline imaging protocols were identical for all volunteers. Dynamic imaging was performed with a T1-weighted fat-suppressed sequence. Enhanced-imaging for a dose of 25 umol/kg was injected into patients' median cubital vein at a flow rate of 2ml/s with a high-pressure syringe. The imaging protocol included sequential acquisition during the arterial phase (20 s), portal venous phase (40 s), and hepatobiliary phase (15-20 min) following contrast administration. Table 1 lists the MRE sequence parameters. All the sequences, except elastrography, were designed to screen for liver and spleen disease.

Multi-frequency harmonic vibrations were generated by the four compressed air-driven pressure pads with 0.6 bar amplitude. The motion encoding was preformed in x, y, and z directions. And the inversion processing was 2D. The wave fields were sampled at eight equally spaced time instances for four mechanical frequencies of 30, 40, 50 and 60 Hz. The motion-encoding gradient (MEG) frequency was 69.44 Hz, and the MEG amplitude was 45 mT/m.

Clinical and other imaging data Collection

The diameter of the portal vein was measured by reader 2 on cantrast-enhanced T1WI axial images in portal venous phase. The measurement point was selected at the point of maximum diameters, located at least 1 cm distant from the confluence of the splenic and superior mesenteric veins and at least 1 cm proximal to the first branch of the main portal vein [1]. Measurement was also taken at the proximal and distal ends of the protal vein. The recorded protal vein diameter was the average of three measurements. The splenic veins was measured by reader 2 in the same manner.

B: Supporting Table and Figure

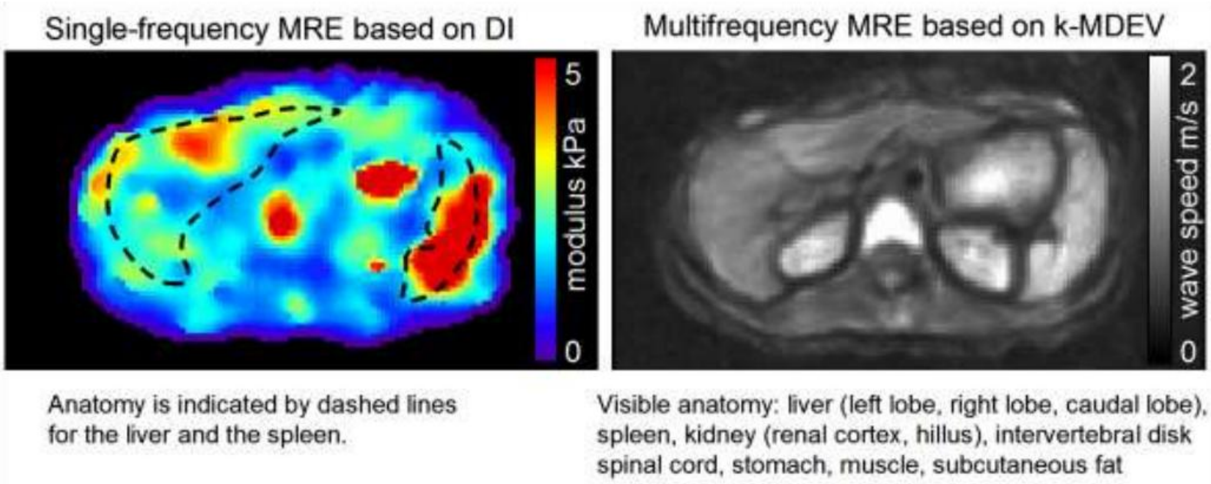

Figure. S1 Comparison of imaging between single-frequency MRE and multi-frequency MRE.

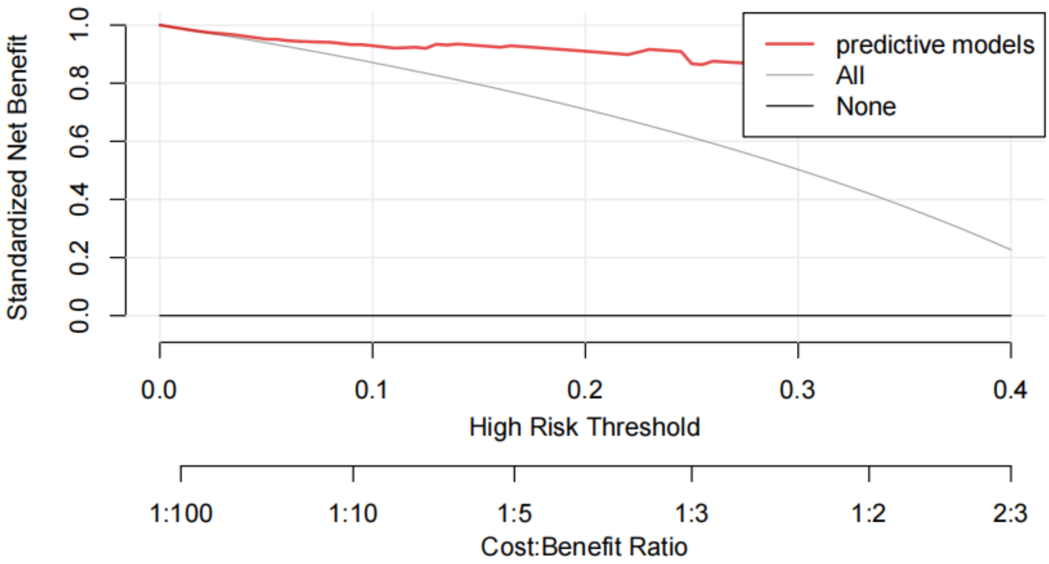

Figure. S2 Decision curve analysis for the predictive model.

$$n \approx \frac{(Z_{1-\alpha/2} + Z_{1-\beta})^2 \cdot (p_1 + p_2)}{(AUC - 0.5)^2}$$

Figure. S3 The formula for sample size calculation. Considering the reported event rate of high-risk esophagogastric varices ranging from 22% to 72% [2,3], the corresponding total sample size would range from 28 to 91, confirming that our study with 54 patients was adequate.

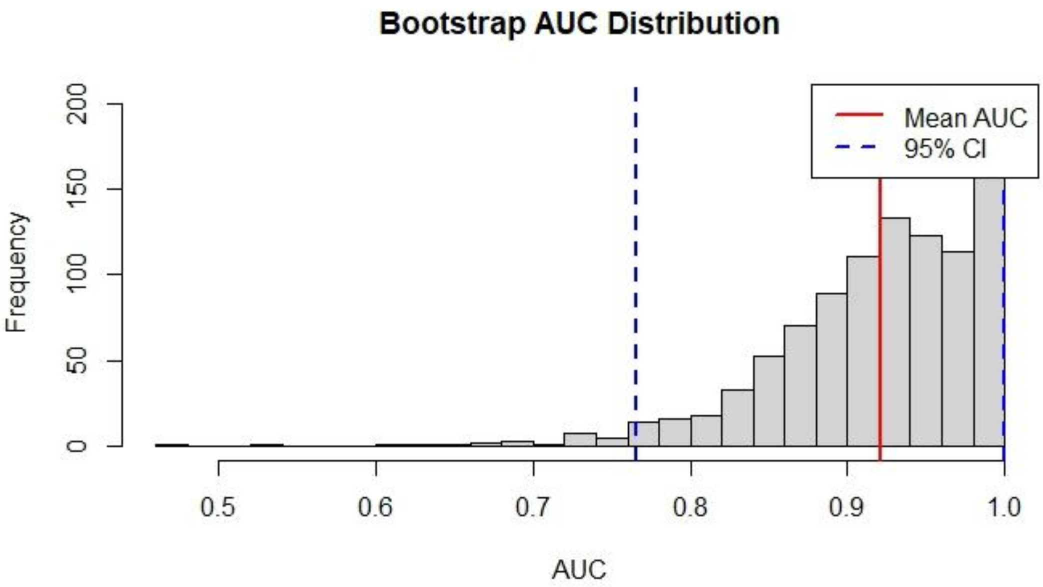

Figure. S4 Bootstrap AUC distribution of the predictive model. The average AUC was 0.92, with a 95% CI of 0.77 - 1.00.

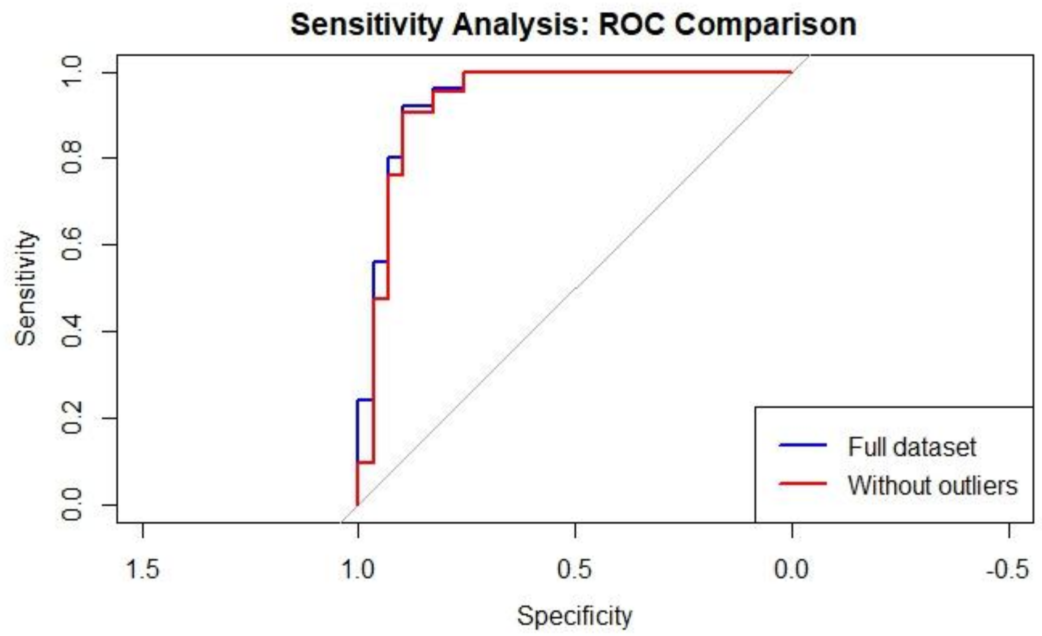

Figure. S5 Sensitivity analysis of the predictive model after excluding outliers. Comparison of ROC curves before (blue) and after (red) excluding extreme outliers of spleen stiffness and portal vein diameter using the interquartile range (IQR) method. The AUC values remained nearly identical (0.94 vs. 0.93), indicating that the model performance was robust and not affected by extreme values.

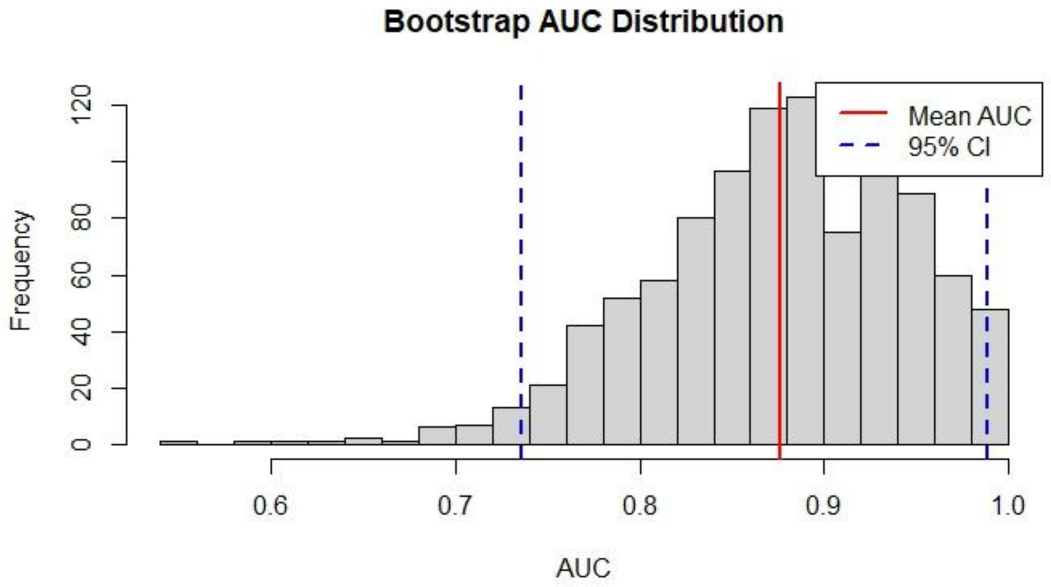

Figure. S6 Bootstrap AUC distribution of guidance based model. The average AUC was 0.88 with a 95% CI of 0.74–0.99.

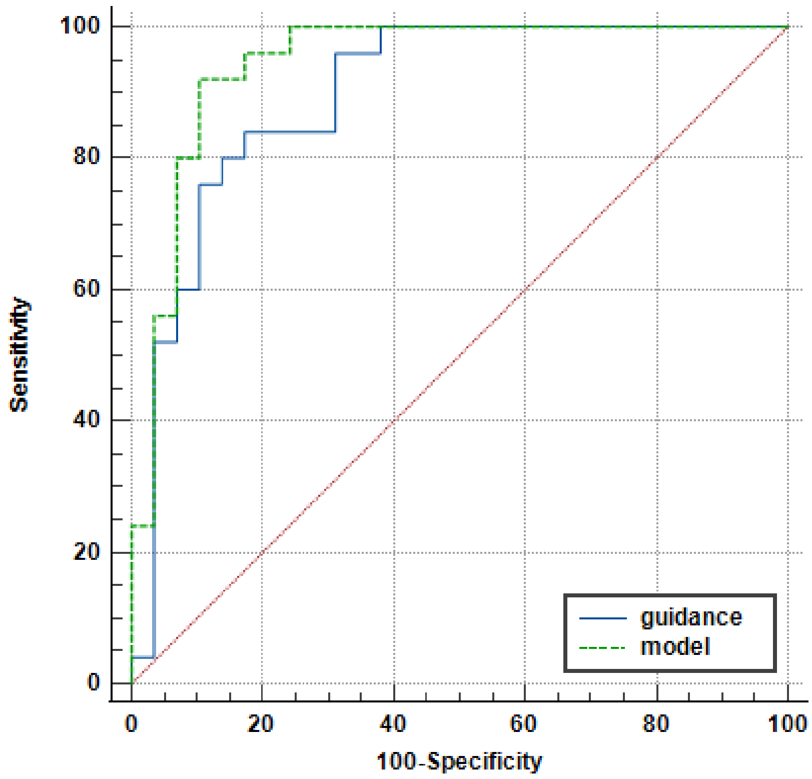

Figure. S7 ROC curve analysis of predictive model and guidance based model. The AUC of the predictive model (AUC = 0.94) shows a tendency to be higher than that of guidance based model (AUC = 0.90). (*P* = 0.30).

1. Stamm ER, Meier JM, Pokharel SS, et al. (2016) Normal main portal vein diameter measured on CT is larger than the widely referenced upper limit of 13 mm. Abdom Radiol (NY) 41: 1931-1936.
2. Ronot M, Lambert S, Elkrief L, et al. (2014) Assessment of portal hypertension and high-risk oesophageal varices with liver and spleen three-dimensional multifrequency MR elastography in liver cirrhosis. Eur Radiol 24: 1394-1402.
3. Abe H, Midorikawa Y, Matsumoto N, et al. (2019) Prediction of esophageal varices by liver and spleen MR elastography. Eur Radiol 29: 6611-6619.
